# An exploratory study on microaggressions in medical school: What are they and why should we care?

**DOI:** 10.1007/s40037-019-0516-3

**Published:** 2019-06-03

**Authors:** Andre Espaillat, Danielle K. Panna, Dianne L. Goede, Matthew J. Gurka, Maureen A. Novak, Zareen Zaidi

**Affiliations:** 1 College of Medicine, University of Florida, Gainesville, FL USA; 2 Department of Pediatrics, Texas Children’s Hospital, Houston, TX USA; 3 Division of General Internal Medicine, Department of Medicine, College of Medicine, University of Florida, Gainesville, FL USA; 4 Division of General Internal Medicine, Department of Medicine, University of Florida, Gainesville, FL USA; 5 Department of Health Outcomes & Biomedical Informatics, University of Florida, Gainesville, FL USA; 6 Department of Pediatrics, University of Florida, Gainesville, FL USA

**Keywords:** Microaggressions, Diversity, Women

## Abstract

**Introduction:**

Microaggressions and their impact have been documented in minority college students; however, little is known about the experience of medical students. This study reports the prevalence and understanding of microaggressions among medical students at the University of Florida College of Medicine (UFCOM), while gaining insights into experiences of medical students dealing with microaggressions.

**Method:**

A nine-question survey was sent out to all medical students at the UFCOM in the spring of 2017 to understand their experiences with microaggressions. The authors used simple statistics and chi-test to analyze the demographic data and an inductive thematic qualitative analysis was performed on the open-ended responses to study medical students’ understanding of the term, experiences, and impact of microaggressions.

**Results:**

The response rate was 64% (*n* = 351/545). Fifty-four percent reported experiencing microaggressions, of those the majority were female students (73% compared with 51% among male students, *p* = 0.0003); for female students from minority backgrounds this was 68% and for white female students 76% (*p* = 0.2606). Microaggressions are more common in the second year of medical school (30%), followed by the third year (23%). Most students were able to recognize and identify microaggressions, but some denied the concept existed, attributing concerns about microaggressions to a culture promoting oversensitivity and political correctness. Students described microaggressions related to sexism; religion; skin colour; and ethnicity. Students described indifference, emotional reactions and denial of the event as coping mechanisms.

**Conclusion:**

Microaggressions are prevalent on a day-to-day basis among medical students with female students from a minority background as well as white female students experiencing more microaggressions. Further research is needed to explore interventions to counter microaggressions in order to ensure a healthy learning environment.

## What this paper adds

Microaggressions are subtle day-to-day put-downs which can impact the wellbeing of individuals. There is a gap in literature regarding the prevalence and experiences of women and minorities with microaggressions in academic medicine. While addressing this gap the study indicates that women are disproportionately impacted by microaggressions. The study results can be utilized by medical educators and institutional leadership to create awareness about microaggressions and to improve learning environments.

## Introduction

The term microaggressions refers to ‘everyday subtle put-downs directed towards a marginalized group which may be verbal or non-verbal and are typically automatic’ [1]. Microaggressions send disparaging messages to individuals because of their perceived group membership. Often the perpetrators of microaggressions are not aware of their actions [1]. In contrast to outright racism, microaggressions are more nebulous, hard to identify and are noted to be a less visible modern form of racism. Microaggressions often occur in the form of unintentional subtle comments, looks or gestures that devalue race or gender [2]. Sue et al. describe microaggressions as cut downs hidden in interpersonal communication or in the environment that convey stereotypes and demean a person’s racial, gender, sexual orientation or heritage [1]. These include comments that are unconsciously disguised as a compliment or a positive statement yet are ‘put-down’ comments. Examples include: telling African Americans that they are a credit to their race, referring to an Asian American as ‘oriental’, telling African Americans that they are loud or Asian Americans that they are too quiet, presuming that a person of colour, specifically African American, is a criminal, assuming a person of colour is janitorial staff or a woman physician is a nurse. These comments often represent metacommunication or hidden messages, which deny the racial, gender, or sexual orientation reality of the marginalized groups. They may leave minorities feeling as if they are not included in the country they are living in and serving or that they are foreign, for example: the assumption that Asian Americans, or Latinos and other racial minorities are foreign-born; the unwillingness to admit to seeing race or a person’s colour with comments such as ‘I don’t see colour when I look at people’ or ‘The most qualified person should get the job, regardless of colour or background’; Implying that people of colour are benefitting from their race through statements like ‘Everyone can succeed in society if they work hard’; Statements made by whites such as ‘I have several black friends’ or ‘I am a woman so I understand your race issues’ are attempts to deny racial bias [1].

Studies cite negative experiences such as gender bias, negative psychological sequelae secondary to overt mistreatment and lack of role models as reasons for the ‘leakiness of the pipeline’—i. e., the departure of students from the path to academia and leadership [3–5]. While overt racism, bullying, and mistreatment of students may be relatively easier to identify, it is harder to identify everyday subtle put-downs or microaggressions. Despite the research on factors contributing to psychological distress in college students, there is little describing medical students’ perceptions and experiences of microaggressions. Using Sue et al.’s definition and categorization of microaggressions as our theoretical framework, we set out to explore medical students experiences with microaggressions, specifically to document:

- The prevalence of microaggressions among medical students at the University of Florida College of Medicine
- Medical students understanding of the term ‘microaggressions’
- The lived experiences of medical students dealing with microaggressions.

## Methods

### Educational setting and respondents

We selected the University of Florida, College of Medicine (UFCOM) for distribution of our survey. Students enrolled in the 4‑year MD program at UFCOM begin with 2 years of system-based didactic learning, with an early introduction to clinical methods and clinical preceptorships before moving on to clinical clerkships. The gender distribution in the MD program ranges from near parity (48% female, 52% males) to parity.

### Data collection

We recruited students during the Spring semester of 2017 via an email invitation posted on the college list-serv that provided them a link to the survey. The survey was open for 3 weeks, and email reminders through the college list-serv were sent weekly until the conclusion of the survey. We obtained permission to conduct the study from the University of Florida Institutional Review Board (IRB number IRB201602183) and informed consent was obtained from all respondents.

### Survey development

As this was an exploratory study, we constructed the survey questions using the literature for guidance. The questions were reviewed by two peers with expertise in medical education, pilot-tested and refined prior to distribution. We used Qualtrics© for distribution and collation of data. The survey consisted of nine questions (Tab. 1). Questions 1–4 related to demographic information while questions 5–6 asked them if they had heard of the term microaggressions and if so to state their understanding of this term. This was then followed by Sue et al.’s definition of microaggressions provided to ensure that students answered the next questions keeping in mind our ‘standard definition’ of microaggressions. The definition was provided for two reasons: 1) As there is a lack of publications on the topic of microaggressions in medical education, it was unclear if research participants would be familiar with the term and 2) To draw attention to differentiating between outright racist comments or acts in contrast to microaggressions, which are subtle. In questions 7–9 they were asked to state if they had experienced microaggressions, and if they had experienced microaggressions to describe the event/s.

**Table 1:** Survey questions

Q1 Gender □ Male □ Female
Q2 Age
Q3 Race/Ethnicity □ American Indian or Alaska Native □ Asian □ Black or African American □ Native Hawaiian or other Pacific Islander □ White □ Hispanic or Latino or Spanish origin □ Not Hispanic or Latino or Spanish origin □ Unknown
Q4 Medical School Classification □ MS1 □ MS2 □ MS3 □ MS4 □ Research Year(s) □ Combined Degree Program
Q5 Have you heard of the term Microaggressions? □ Yes □ No
Q6 If you have heard of the term ‘Microaggressions’, what is your understanding of the term?
Q7 ‘Microaggressions are the brief and commonplace daily verbal, behavioral, and environmental indignities, whether intentional or unintentional, that communicate hostile, derogatory, or negative racial, gender, sexual-orientation, and religious slights and insults to the target person or group. Perpetrators are usually unaware that they have engaged in an exchange that demeans the recipient of the communication.’ Taken from: ‘Microaggressions in Everyday Life. Race, Gender, and Sexual Orientation’ by Derald Wing Sue. Keeping in mind the above definition have you ever experienced Microaggressions during your time in Medical School? □ Yes, during medical school □ Yes, during my post graduate education, but before medical school □ Yes, during my undergraduate training □ Yes, but not in an educational setting □ No
Q8 If ‘yes’, in less than 150 words, please describe the event and how it made you feel.
Q9 If you selected ‘Yes, during medical school’, please indicate the year of study when this occurred. □ Didactic Years □ Required third year clerkships □ Away rotations □ Fourth year clinical rotations

### Analytic procedures

D. P. and A. E. initially coded the data using Braun and Clarke’s framework for thematic analysis [6]. Following Braun and Clarke’s methodology, D. P. and A. E. independently analyzed responses, highlighting material of interest and annotating them with marginal comments. They generated codes and categorized them into themes based on patterned responses and the richness of responses. An inductive thematic analysis approach was used, which is a process of coding the data without trying to fit it into a pre-existing coding frame, or the researcher’s analytic preconceptions. In the inductive approach, the themes identified are strongly linked to the data themselves. In keeping with critical research practice [7], we employed critical reflexivity to explore our positions on the dataset. We used a combination of in-person meetings and phone calls to discuss our perspectives on the data based on our individual backgrounds; we discussed implicit bias and documented differences in opinions that may have impacted data analysis. An audit trail was maintained consisting of analysis of team meetings, discussions of codes, revisions and team member comments. Member check was done by inviting a respondent to review themes and affirm that the themes reflected participants’ experiences. Simple statistics were used to describe the demographic data, and chi-squared tests were used to analyze the associations between gender and microaggression experiences.

## Results

Of the 545 students at UFCOM, 351 agreed to participate (64%); however, some participants did not answer all the questions. Respondents consisted of 61% females and 39% males. The average age of respondents was 25 (range 18–45 years). Of the respondents, 63% identified racially and ethnically as ‘White’, 11% as ‘Asian’, 10% as ‘Black or African American’, 9% as ‘Hispanic or Latino or Spanish origin’, 7% as ‘Unknown’. First-year medical students comprised 22% of the respondents, 2nd year-medical students 30%, 3rd year medical students 24%, 4th year medical students 22%, and combined degree students (MD PhD, MD MPH) comprised 2%.

Of the respondents, 56% had heard of the term microaggressions while 44% had not heard the term. A total of 54% reported experiencing microaggressions in medical school and 10% in settings other than medical school and 36% reported no microaggressions. Out of the students who experienced microaggressions in medical school, 50% noted that they experienced microaggressions in their preclinical years, while 46% noted experience in their clinical years and 4% in elective rotations.

Of the females 73% experienced microaggressions. Chi-squared tests were used to analyze the associations between gender and microaggression experiences (Tab. 2). For *overall experiences with microaggressions, including instances outside of medical school*, females were more likely to experience microaggressions (73% compared with 51% among males, *p* = 0.0003). Regarding *microaggressions in medical school only*, females were again more likely to encounter microaggressions (60% compared with 45% for male students, *p* = 0.0206). Among female students, 68% from minority backgrounds and 76% of white female students experienced microaggressions (Tab. 3), including instances outside of medical school (*p* = 0.2606).

**Table 2:** Comparison of gender differences in experiences with microaggressions

	males (*n* = 1)	females (*n* = 159)	chi-Squared *p*-value
	*n* (%)	*n* (%)	
experience with microaggressions:			
– overall	53 (51.0%)	116 (73.0%)	0.0003
– in medical school	47 (45.2%)	95 (59.8%)	0.0206

**Table 3:** Comparison of ethnicity differences in experiences with microaggressions among females

	experience with microaggressions	
	overall	medical school
	*n* (%)	*n* (%)
nonwhite (*n* = 59)	40 (68%)	34 (58%)
white (*n* = 100)	76 (76%)	61 (61%)
chi-square *p* value	0.2606	0.6752

The analysis of the students’ responses to the question asking them to state their understanding of the term ‘microaggressions’ revealed four main themes and additional sub-themes: 1) They attempted to provide definitions of the terms which we describe in the theme Definitions of microaggressions; 2) They described the consequences of microaggressions over time: Long-term impact; 3) The impact on society: Microaggressions as a societal issue; 4) Some denied the existence of the phenomenon: Denial of concept.

## Definition of microaggressions

Students understanding of ‘microaggressions’ as a definition were grouped into three further sub-themes: 1) Subtle or inadvertent comments or actions; 2) Passive aggressive behaviour; 3) Use of the term in a literal sense: small aggression. All responses attempted to define the term but were different in their focus on how the aggression is distributed or perceived by an individual.

### Subtle or inadvertent actions

Respondents focused their definition of microaggressions on how the aggressor creates ‘subtle’ or ‘inadvertent’ distress to the recipient. Other terms that students used to address this concept included ‘accidental’, or ‘inconsequential’ actions towards another individual. Responses also noted that the perceived aggression individuals felt was not the intention of the aggressor. The aggressor has no intention of ‘aggressing another but is perceived as doing so’.

*To me, a microaggression is a form of interpersonal aggression that involves the use of words or actions that on their surface may not appear to be intended to harm others but may end up doing so depending on the perceptions of the receiver. In other words, a microaggression is a seemingly innocuous word or action that functions on a less conscious level to harm others.*

### Passive aggressiveness

A subset of responses likened microaggressions to passive aggressiveness. All responses noted that these two terms were either ‘similar’, ‘somewhat like’ or ‘one and the same’. These responses also included a relationship between an aggressor and recipient that invoked a negative response directed to the recipient.

*Small inappropriate remarks or passive aggressive actions towards a person involving work or personal life.*

### Small acts of aggression

Respondents also defined microaggressions in a literal sense. These responses equated ‘micro’ to ‘small’ and thus created definitions of ‘small’ aggression. While they did not give examples of what constitute ‘small’ aggressions, some responses noted the aggression that ‘targets a minority’. Other responses also noted that these acts can ‘go under the radar’ or may be ‘hard to call out’.

### Long-term impact

Responses were also noted to focus on the effects of microaggressions over a period of time. Respondents focused on how these aggressions can build up to cause ‘distress’ or impact the ‘victim’s well-being’. In addition, responses noted underlying subtlety, repetition, and impact of the intention. One respondent illustrated differences based on the reference point of aggression, stating that these acts may seem ‘relatively neutral alone’, but can ‘cause serious distress over time’.

*Minor acts of offense that individually do not cause significant distress yet when aggregated over a period of time has a negative effect psychologically. Often the acts are believed to be linked to a person’s social or physical identity.*

### Microaggressions as a societal issue

Respondents noted that microaggressions were not solely caused by interactions of people, but rather reflected larger issues present in society. These acts were noted to ‘propagate larger systems of violence and oppression’ and indicated larger scale problems in society.

*Seemingly small comments or actions made that reflect a larger societal issue of sexism, racism, homophobia, etc. These comments ‘seem’ harmless (micro) but are harmful to the recipient.*

### Denial of concept

A subset of respondents denied the concept of microaggressions. Responses illustrated that those receiving these aggressions are ‘extremely sensitive individuals’. Others noted that the term was made up and ‘used by people who have never felt real oppression’. One respondent explained that:

*Microaggressions are a way of perpetuating the culture of victimhood that plagues our society and polluting the minds of today’s over privileged, faux social justice warrior youth.*

While another noted it was a way to ‘crack down on free speech’. Several responses described how microaggressions were either made up or the recipient was overly sensitive.

*Extremely sensitive individuals use this word to describe comments that intentionally or unintentionally offend their fragile egos because they have been accustomed to receiving participation trophies in little league and subsequently have not developed the proper coping mechanisms when encountering someone who doesn’t like them, disagrees with their views, or generally has an opinion different from their own. It’s a major development in the so-called ‘PC culture’. Using words like microaggression enables the user to declare oneself ‘victimized’ in an intellectual way.*

Responses also highlighted that acknowledgment of this term was unhealthy and that this was a ‘new term to keep academics employed’. One respondent noted that ‘embracing the concept of microaggressions is an unhealthy and childlike response’, while another respondent noted that it was a term to ‘keep … the people divided’.

*To have suffered a microaggression, you must first adopt the ‘woe is me’ attitude of a perpetual victim who intends to feel threatened by every well-intentioned word, phrase, or action that you may encounter on a daily basis.*

Respondents were also asked to describe their experiences with microaggressions. Three main themes emerged from this data: 1) Experiences related to sexism; 2) Experiences related to social-cultural issues; 3) Reactions to microaggressions.

### Sexism

Respondents reflected on experiences of microaggressions in three different settings which emerged as sub-themes: Preclinical; Clinical—Surgery; and Clinical—General. These individuals drew from their experiences in these settings to illustrate when microaggressions took the form of sexism.

### Pre-clinical

These responses looked at experiences that were not specific to the clinical setting. Respondents noted microaggressions in the classroom, during team-based learning sessions, and during simulated clinical scenarios in the first two years of the curriculum. One student noted how they were ‘ridiculed for wrong answers’ in ‘more stringent ways than my classmates’ of different gender.

*Male faculty has also shown ‘inappropriate dress’ pictures, and they only featured women. This made me feel upset that only women could break the dress code.*

### Clinical—surgery

Respondents described comments related to childbearing, domestic relationships and obligations that would negatively impact their decision to pursue surgery. One respondent noted that she was told: *‘Any woman in surgery will be lonely and miserable because their husband will leave them/cheat on them because they can’t be expected to not cheat when their wife is so busy’*. Others were told that they should avoid surgery because they would not be able to be a ‘good mother’, ‘have babies’ and ‘tolerate the work week’ at the same time. Another respondent explained how seemingly everyday comments that had good intentions, were actually perceived in an opposite manner.

*In my time on surgery, I was referred to solely in seemingly disparaging terms such as ‘sweetheart’ in the OR by a young male attending. As a female interested in surgery, I felt extremely frustrated that I was not being taken seriously and felt very belittled by the experience.*

### Clinical—general

These responses included those that noted experiences in the clinical setting but did not mention a surgical setting. One student noted how an attending made her feel like she was being viewed ‘different from my male colleagues’ and insinuated that she was trying to use her ‘gender and sexuality as a means of success’. Other responses looked at how others could not comprehend there was a female in the medical field. Specifically, being ‘mistaken for a nurse’, and not being ‘taken as seriously as my male counterparts’. Others note that male domination in specific fields may lead to a lack of success or ‘guilt’ for pursuing these fields. Overall, responses explored the difficulty that medical students who identify as woman experience in the medical field as compared with their male counterparts.

*Being called various things like ‘princess,’ ‘sweetheart,’ ‘nurse,’ ‘darling,’ etc. by patients, residents, or attendings. Comments about how I look or dress. Some men probably assume they’re complimenting me when it’s actually belittling. It’s impossible to be a woman in medicine without being reminded you’re a woman every day, most of the time without people realizing they’re reminding you.*

### Social cultural

These responses highlighted different experiences that related to social-cultural interactions. Specifically, how individuals’ responses focused on experiences of Religion; Colour of Skin; Ethnicity; and Sexual Orientation. These respondents noted how differences in these beliefs led to microaggressions.

### Religion

Students discussed experiences of microaggressions as related to their religious beliefs. One student noted how members of the medical team mocked ‘people who attend church and participate in religious activities’. While another noted that classmates spoke ‘generally’ and could not understand ‘how anyone in medicine could also be very religious or believe in God’. Students expressed that it was difficult to have religious beliefs or to be an atheist due to the appearance of religion in schoolwork.

*Numerous circumstances where people made me feel like it was impossible to be a pro-life, Christian, Republican physician while remaining rational, having thoughtful discussions with other professionals, and treating all patients with respect and compassion.*

*I am an atheist, and there are a lot of religious comments and assumptions made by both students and faculty that all in the class are believers, frequently excluding those of us (>20% of the nation) who are not.*

### The colour of skin

Students noted the effect the colour of their skin had on their experience of microaggressions. A student noted a lack of communication by the team after a patient used the *‘n-word’ which made them feel like ‘what the patient called me was okay and they didn’t care’*. Another student commented that due to their race, they were assumed to have ‘more black friends’. Students also experienced microaggressions because they identified as white. One respondent noted that they *‘had a Hispanic attending who was joking with a Hispanic medical student about how she would ‘never fall for a guy like [me] because [I am] white’.’*

*I was having a conversation with a faculty member who said since there has been more diversity in the medical school students at UF board exam scores have declined. We were in the middle of a conversation regarding students’ performance on Shelf exams. I felt like I was part of that reason for lower scores, that I was incapable of matching up to the majority of my class which is white.*

### Ethnicity

Students also noted that differences in ethnicity and beliefs led to experiences with microaggressions. Respondents noted how others assume their ethnicity based on their appearances such as an assumption that a student was Chinese, and a professor asking about the history of ‘Japanese internment’ when they were not Japanese. Another student noted a common occurrence of microaggression was the ‘mention of the Colombian cocaine cartel’ when others learn of their Colombian descent. Another student commented, *‘My roommate’s boyfriend asked if Turkish people believe in female genital mutilation’*.

### Sexual orientation

Respondents also drew from experiences of microaggressions due to their sexual orientation. These students noted ‘inappropriate questions’ or ‘confused looks’ after ‘displaying affection with a person of my same gender’. In the classroom, a fellow classmate asked if parents’ ‘dysfunctional relationship caused’ individuals to ‘identify as LGBT’. Other students have experienced clinical scenarios where the ‘clinical staff referred to LGBT people as ‘people like that’.’

*Our school solely presents gay men in question stems in order to present a question regarding HIV/AIDS. This has made me feel unsafe. We have never been presented with a gay woman, which has made me feel invisible.*

### Reaction to microaggressions

Respondents described their reactions to the microaggressions. The reactions are categorized as two sub-themes: indifference to the event and emotional response.

### Indifference

Students noted that microaggressions they experienced were not something to be concerned about. Specifically, they would try to ‘forget about the incident’, while others assumed ‘the other person was being ignorant and not actually hateful or mean’. All in all, these students tried not to think about such events.

*I don’t really care. I don’t let it affect me. I may consider why it is inappropriate or why that word used in that context is offensive. I use it as an opportunity to grow.*

*It often made me feel uncomfortable but never to the point where I felt like I needed to report faculty/peers. I would often just try to forget about the incident*.

### Emotional

Students also had a negative, emotional response to microaggressions. One respondent noted that they felt ‘singled out and embarrassed’. Another student felt ‘powerless’ and ‘Shitty’: *‘It made me lose confidence in myself for a while and it was really upsetting’ *and* ‘It made me feel like I was a bad person for the values that I hold’.*

## Discussion

### Principal findings

Our research shows that the majority of students at UFCOM (56%) are aware of the term microaggressions. While 54% of respondents report having experienced microaggressions, female students disproportionately experience more microaggressions compared with males, and among females, students from minority backgrounds *as well* as white female students experience microaggressions. Microaggressions are more common in 2nd-year medical students followed by 3rd-year medical students. Students are familiar with the term microaggressions and the long term and societal impact; however, there are some who denied the concept. Students described microaggressions related to sexism, religion, skin colour, and ethnicity. Despite providing a definition of microaggressions in the survey, a few examples provided by students of ‘microaggressions’ are actually examples of outright racism or sexism, which can be categorized as ‘microassaults’ according to the framework by Sue et al., which we employed for this study [2]. As coping mechanism students described indifference, emotional reactions and denial of the event. While literature about outright racism is easy to recognize and call-out, and data on student mistreatment have been published, to our knowledge this is the first paper describing subtle put-downs (microaggressions) which are harder to recognize.

### Relationship to literature

Isolated microaggressions might be dismissed as unfortunate events and thought not to have a detrimental impact on the individual; however, when these experiences are common they impact self-esteem, and result in anger and frustration [1]. Victims of microaggressions may develop maladaptive behaviours including depression, hypervigilance, scepticism, rage, anger, fatigue and hopelessness [1, 8]. It is of note that our results indicate that women irrespective of the racial background are more affected by microaggressions. Such cumulative effects of microaggressions can lead to the well-documented ‘leaky pipeline’ for women and minorities particularly in academic medicine [9]. Female medical students have been documented to become desensitized to inappropriate behaviours and note that they were ‘just too tired to care’ [10].

The number of minorities who have successfully traversed the pipeline into the academic medical profession is also sparse. Minorities comprise only 4% of medical school faculty positions nationally [11]. Our results note that minority groups experience microaggressions, based on race, religion, ethnicity and sexual orientation, which can have a lasting impact. In our study female students are disproportionately impacted and females of all racial ethnicities Hispanic, Latino or Spanish, Asian as well as White students experienced microaggressions.

We postulate that experiencing microaggressions earlier in education likely leads to loss of empathy and cynicism noted in later years. It is also possible that cynicism may have prevented more 3rd and 4th year medical students from responding to our survey. Examples provided in our survey by students in the pre-clinical years (i. e. inappropriate dress pictures only featuring women) indicate that educators need to ensure the curriculum being taught, as well as the hidden curriculum, promotes inclusiveness in the pre-clinical as well as clinical years.

Though we were not able to find literature in medical education regarding the denial of the concept of microaggressions, we point out that statements made by students regarding microaggressions promoting a ‘PC culture’ (politically correct) or ‘faux social justice warriors’ mirror the current conversations in the news media. Steps need to be taken at institutional and national levels to engage groups in conversations in an effort to create a better understanding of social issues.

## Limitations and strengths

We failed to include broader options for participants regarding gender identity and sexual orientation in our demographic data questions on the survey, inadvertently committing a microaggression. Two respondents pointed this out to us in their comments on the survey. We used this experience to ensure future surveys sent out included the additional options. It is possible that if the option had been provided, more students, with different gender identities and sexual orientation, would have shared their experience. Our inadvertent microaggression is similar to that documented by others noting that having awareness of microaggressions, minority issues, and implicit biases does not shield us from committing microaggressions [12]. Our results are based on self-report data from a single institution and could have been impacted by student interest in completing the study. We also did not specifically look into actions taken by students, i. e. reporting of these incidents. The strengths of the study include a good response rate and the detailed narrative experiences that were shared. We feel that the results of this study are fairly generalizable and can be used by other schools to plan interventions to counter microaggressions.

## Learning and implications

The results of our study have several learning points and implications for educators and institutions. At our institution, the study resulted in an effort to develop an online faculty development module focusing on diversity and inclusion. Providing ‘*counter space*’ which are intentionally created networks/safe spaces or sanctuaries where participants can develop relationships and support for coping with microaggressions have been described as essential for the academic success of minority students. The Diversity Office at UFCOM provides support to such ‘counter space’ in the form of minority group meetings with active faculty from minority backgrounds. Ensuring such social support has been shown to be an important factor in determining academic success [13]. While an ‘avoidant coping mechanism’ may exacerbate stress [14], verbalizing the issue or confronting individuals is described as a ‘Catch-22’ dilemma or ‘damned if you do and damned if you don’t’ situation by Sue et al. [2]. Conveying a message using humour has been described [15] and may be particularly useful with inter-racial dialogue training. Formal mentoring programs have also been shown to result in greater progress of minority mentees [16]. Becoming involved in social action, such as joining a group, researching and petitioning leadership to create awareness are positive coping strategies [17]. Mindfulness practice, spirituality, prayer, and rituals can also be used to cope with frustrations brought up by microaggressions [18–20]. Additionally, social media movements such as #ILookLikeASurgeon and #BlackMenInMedicine have been used to overcome stereotypes and promote inclusion in medicine and surgery. The Implicit Association Test (IAT, available free online) which measures attitudes and beliefs that people may be unwilling or unable to report is a tool that can be used to create awareness of biases [21]. The faculty at UFCOM are also being provided with implicit bias training opportunities. Such exercises should be followed by debriefing meetings and advanced training opportunities. However, studies using the IAT tests have shown implicit attitudes are developed over a long period of repeated exposure and are difficult to change [22]. Finally, institutions should develop longitudinal curricula that focus on diversity training utilizing multiple teaching formats [23]. Further research is needed to explore the impact of microaggressions on student burnout, anxiety, depression and desire to pursue academic medicine.
